# Doxorubicin Conjugated γ-Globulin Functionalised Gold Nanoparticles: A pH-Responsive Bioinspired Nanoconjugate Approach for Advanced Chemotherapeutics

**DOI:** 10.3390/pharmaceutics16020208

**Published:** 2024-01-31

**Authors:** Gaurav Chauhan, Vianni Chopra, América García Alvarado, Jocelyn Alexandra Gómez Siono, Marc J. Madou, Sergio Omar Martinez-Chapa, Manish M. Kulkarni

**Affiliations:** 1 School of Engineering and Sciences, Tecnologico de Monterrey, Av. Eugenio Garza Sada 2501 Sur, Monterrey 64849, NL, Mexico; vchopra@tec.mx (V.C.); amealvarado-@hotmail.com (A.G.A.); mmadou@uci.edu (M.J.M.); smart@tec.mx (S.O.M.-C.); 2 Department of Mechanical and Aerospace Engineering, University of California Irvine, Irvine, CA 92697, USA; 3 Centre for Nanoscience, Indian Institute of Technology Kanpur, Kanpur 208016, India

**Keywords:** gold nanoparticles, hard protein corona, γ-Globulin, doxorubicin, nanobioconjugate, pH responsive, anticancer hybrid

## Abstract

Developing successful nanomedicine hinges on regulating nanoparticle surface interactions within biological systems, particularly in intravenous nanotherapeutics. We harnessed the surface interactions of gold nanoparticles (AuNPs) with serum proteins, incorporating a γ-globulin (γG) hard surface corona and chemically conjugating Doxorubicin to create an innovative hybrid anticancer nanobioconjugate, Dox-γG-AuNPs. γG (with an isoelectric point of ~7.2) enhances cellular uptake and exhibits pH-sensitive behaviour, favouring targeted cancer cell drug delivery. In cell line studies, Dox-γG-AuNPs demonstrated a 10-fold higher cytotoxic potency compared to equivalent doxorubicin concentrations, with drug release favoured at pH 5.5 due to the γ-globulin corona’s inherent pH sensitivity. This bioinspired approach presents a novel strategy for designing hybrid anticancer therapeutics. Our study also explored the intricacies of the p53-mediated ROS pathway’s role in regulating cell fate, including apoptosis and necrosis, in response to these treatments. The pathway’s delicate balance of ROS emerged as a critical determinant, warranting further investigation to elucidate its mechanisms and implications. Overall, leveraging the robust γ-globulin protein corona on AuNPs enhances biostability in harsh serum conditions, augments anticancer potential within pH-sensitive environments, and opens promising avenues for bioinspired drug delivery and the design of novel anticancer hybrids with precise targeting capabilities.

## 1. Introduction

Noble metal nanoparticles (Au, Ag and Pt) have found an important role in biomedical applications like nanomedicine, chemical and biosensing [1,2,3]. AuNPs display a wide array of optical, photothermal (localised surface plasmon resonance, LSPR) properties, controllable surface and size modifications (nanorods, stars etc.) and a relatively facile synthesis [4,5,6]. Ultra-small AuNPs have shown efficient binding efficiency in tumour zones, due to the EPR effect (“enhanced permeability and retention effect”), thus enabling them to have longer systemic circulation times. This makes them the most popular noble metal platform in nanochemo therapeutics.

There are some studies which report the toxic effects of AuNPs, which can be attributed to the small size and surface release of free ligands [7]. When gold nanoparticles come into contact with various plasma proteins and other biomolecules, there is a surface binding phenomenon called “protein corona” that occurs [8]. Depending upon the physicochemical characteristics, the degree of protein corona formation varies. This corona may affect the organ and cellular distributions of these nanoparticles, as well as their in vivo toxicity thus eliciting different immunological responses [9,10,11,12].

Exploitation of this interaction can be used to precoat gold nanoparticles with different biomolecules, resulting in biomimicking core-shell systems [11,13]. Chen et al. have formulated bovine serum albumin stabilised gold nanoparticles conjugated with Methionine and drug for a prodrug approach [14]. Wójcik et al. formulated glutathione-stabilised gold nanoparticles non-covalently modified with doxorubicin [13] and Licciardi and coworkers formulated inulin-coated gold nanoparticles for selective delivery of doxorubicin [15].

The physiochemical characteristics of AuNPs and the nature of interaction with the external environment determine the fate of their conjugation with biomolecules and ligands. There are three basic mechanisms via which biomolecules become conjugated to noble metal nanoparticles viz. electrostatic interactions (e.g., adsorption of negative-charged DNA to positive-charged gold NP), chemisorption (e.g., quasi-covalent binding of thiol-functionalised biomolecule to gold NP), affinity-based (e.g., His-tag protein binding to Ni-NTA derivatised gold NP). Gold nanoparticles have been conjugated using the above techniques with immunoglobulins, serum albumins, streptavidin, DNA and other proteins [16,17,18]. 

γ-globulin is a human serum protein, named according to its separation position in serum protein electrophoresis. It belongs to the family of globulins, has a molecular weight of approximately 155–160 kDa and is made up of two heavy chains, each of about 55–60 kDa and two light chains, each of about 25–28 kDa. Globulins have not been extensively utilised for the stabilisation and protein coating of gold nanoparticles. Mirshafiee and coworkers studied protein corona formation and cellular uptake by pre-coating silica nanoparticles with human serum albumin and gamma globulin [19]. They have shown that gamma-globulin pre-coating promotes the recruitment of opsonins, namely, immunoglobulins and complement components (e.g., complement C3, C4A, C5, C1s, C8 subunits, C7, and C6) in the plasma to the protein corona after 1 h of incubation. Although, due to the nonspecific binding of other proteins, the opsonin effects were diminished and there was no increase in uptake by raw 264.7 macrophage cells [10]. The isoelectric point “pI” of γ-globulin (pI ~7.2) plays a key role in its selection in this nanobioconjugate approach. According to the literature, environmental pH affects the net charge on the molecular surface and a comparatively positive or negative charge can be observed due to the gain or loss of protons, respectively.

Doxorubicin (Dox) belongs to the anthracycline category of antineoplastic agents. It is also the most widely used chemotherapeutic drug for different types of human tumors: prostate, breast, ovarian, pancreatic etc. However, the limitations of DOX therapy, such as hypersensitivity reactions, liver and kidney dysfunction and severe cardiotoxicity have turned the tables towards different drug delivery systems. Systems like liposomes (doxorubicin-loaded PEGylated liposomes DOXIL^®^), micelles Genexol-PM (PEG–PLA micelles), carbon nanotubes, drug conjugates and PEGylated nanoparticles, (PEG–polyaspartic micelles) are examples [20,21]. All these nanomedicines have not yet shown exemplary results in clinical applications. In a recent work carried out by Banu and co-workers, gold nanoparticles loaded with doxorubicin were functionalised with folic acid to achieve selectivity towards MDA-MB-231 breast cancer cells [22]. These systems which involve both gold and drug have shown synergistic effects in terms of cell death and also better cellular uptake [23].

Hybrid therapeutic drug carriers with exceptional biological compatibility and inherent potentiation effects are the need of current medical research. Specifically, in the area of chemotherapeutics, AuNP-based nanosystems could provide potentiating effects owing to the inherent anticancer properties of AuNPs [24,25,26]. AuNPs are capable of altering the cell cycle including cell division, signalling, and proliferation. DNA damage, cytokinesis arrest, and programmed cancer cell death were also reported in support of the anticancer ability of AuNPs [2,27,28]. 

**Scheme 1:**
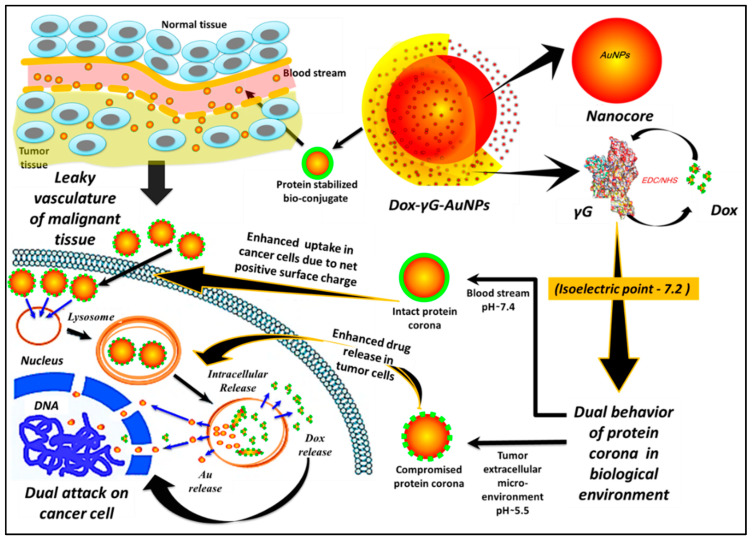
Biostable nanohybrid (nanobioconjugate) strategy aiming multiple targets concomitantly, to provide potentiating anticancer effects.

In this work, we designed γ-globulin functionalised gold nanocore (AuNPs < 20 nm) as an efficient drug carrier and hybrid nanosystem aimed to provide potentiating effects in cancer cells. The model anticancer drug, Dox, was chemically tethered to the hard γ-globulin corona on the nanogold core providing a bioinspired core shell system. According to our hypothesis, γ-globulin functionalised AuNPs will serve as a biostable delivery vehicle, controlling the exposure of tethered drug molecules in a cancer cell microenvironment. Moreover, concurrent exposure of AuNPs along with Dox will provide synergising effects. Further, γ-globulin (pI ~7.2) will possess a comparatively positive charge at lower pH values (i.e., pH ~6.5, the extracellular tumor microenvironment), which will eventually assist cellular uptake in cancer cells. γ-globulin solubility is highly pH-sensitive; they are least soluble at pH = pI but will be much more soluble at pH = pI ± 1.2. This exceptional property of pH-responsive solubility will help in designing this nanobioconjugate approach allowing for a burst release of conjugated drug within a tumor microenvironment [29,30]. Scheme 1 shows a cartoon representing this nanobioconjugate strategy.

## 2. Material and Methods

Gold (III) chloride trihydrate (HAuCl_4_), gamma globulin and doxorubicin were purchased from Sigma-Aldrich (Bangalore, India). Deionized water (Millipore Milli-Q grade, Merck, Darmstadt, Germany) with a resistivity of 18.2 MΩ was used in all the experiments. The rest of the chemicals and materials used in the study were commercially available unless otherwise specified and were used without further purification. 

### 2.1. Synthesis of γ-Globulin (γG) Stabilized Gold Nanoparticles (γG-AuNPs)

The Turkevich synthesis method was adopted using trisodium citrate as a reduction/stabilising agent to synthesise AuNPs. Briefly, 90 mL of 0.5 mM chloroauric acid (HAuCl_4_) was heated until the sample started boiling and then was immediately reduced with 10 mL of 38.8 mM trisodium citrate (Na_3_C_6_H_5_O_7_) under controlled stirring conditions (500 rpm). Heating was stopped after a pale-violet colour became evident and the solution was further stirred until a blood-red-coloured suspension of citrate stabilised AuNPs was obtained. A total of 29 mL of AuNPs nanosuspension (500 µg/mL) was then incubated with l ml of 6.25 µM γ-Gl solution in double distilled water. The reaction was allowed to proceed for 30 min at ambient temperature and the resultant protein-coated as well as protein -tabilised gold nanoparticles were obtained. 

### 2.2. Quantification Tightly Bound Hard (γG) Corona: Standard Bicinchoninic Acid “BCA” Assay

A tightly bound hard γ-globulin corona layer on the AuNPs was estimated by quantifying this protein around the nanometal surface [1]. The obtained nanosuspension was then centrifuged for 1 h at 15,000 rpm and the supernatant left after centrifugation was subjected to the quantification of unconjugated γ-globulin. A standard bicinchoninic acid (BCA) assay was performed for the quantitative analysis of free protein at 562 nm using a UV–Vis spectrophotometer and a subtraction method was used (Equation (1)) to quantify hard γ-globulin corona.

γG _hard corona_ = γG _total_ − γG _supernatant_ (1)

### 2.3. Synthesis of Dox Conjugated γG-AuNPs, i.e., “Dox-γG-AuNPs”

**Scheme 2:**
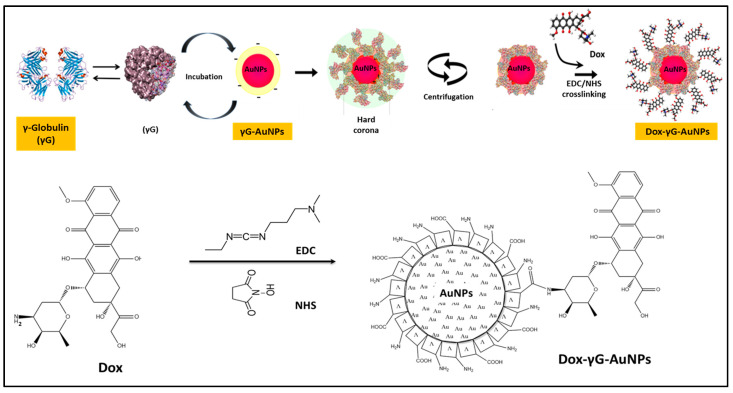
Synthesis of γ-globulin stabilised AuNPs, and chemical conjugation of doxorubicin to the hard corona of the protein deposited on these γG-AuNPs.

Dox-γ-Gl-AuNPs nanobioconjugate was synthesised by using 1-Ethyl-3-(3-dimethylaminopropyl)carbodiimide (EDC) and N-Hydroxysuccinimide (NHS)-based crosslinking chemistry [31]. Firstly, the linker was attached to the surface-exposed carboxylic groups of doxorubicin and then the crosslinking was performed with exposed amino groups of hard γ-globulin corona on the AuNPs surface. The synthetic scheme for the preparation of Dox-γG-AuNPs nanobiocomposite is displayed in Scheme 2. For initial activation of the drug, 4.5 mg EDC and 1.7 mg NHS was added to 20 mL of 0.2 mg/mL Dox solution in deionised water in a centrifuge tube. This tube was incubated while shaking it constantly for 2 h. A total of 80 mL of γG-AuNPs (650 µg/mL) suspension was then added to the tube and again the tube was rotated overnight. Finally, the obtained Dox-γG-AuNPs was purified by dialysis (using 1000 molecular weight cut off against PBS buffer, pH ~7.2) to remove the non-conjugated Dox. Finally, the nanobioconjugate was recovered by centrifugation at 15,000 rpm for 20 min and the yield was calculated after drying at ambient temperature in the dark. Dox-γG-AuNPs nanobioconjugate was then characterised for various physicochemical aspects.

Dox-γG-AuNPs were further evaluated for Dox loading per mg of the complex. Dox content was analysed using the subtraction method during the conjugation procedure. The unbound Dox was further analysed at λ-max 480 nm using a UV spectrophotometer (Varian Cary 50 Bio spectrophotometer, Agilent, Santa Clara, CA, USA), referring to a calibration curve prepared under the same condition. The loading efficiency of Dox in the nanobioconjugate was calculated spectrophotometrically using Equation (2).

% Loading Efficiency = (Weight of loaded Dox − Weight of free Dox) × 100/Weight of loaded Dox(2)

### 2.4. Characterisation

Synthesised nanobiocojugates were characterised for particle size, polydispersity index (PDI), zeta potential using Zeta sizer (DLS 4C Beckman Coulter, Seto, Japan), UV–VIS spectroscopy (using Varian Cary 50 Bio spectrophotometer, Agilent, Santa Clara, CA, USA), FT-IR Spectrophotometer (Perkin Elmer, Waltham, MA, USA), and X-ray diffraction (XRD, PAN analytical, Kassel, Germany, performed from 5° to 80° at a scanning speed of 2° per minute) to determine the crystal structure. X-ray photoelectron spectra (XPS, PHI 5000 Versa Prob II, FEI, Topsfield, MA, USA) were measured in the binding energy range from 0 to 1100 eV. Morphological characterisation was performed by using transmission electron microscopy (TEM; FEI Titan G2 60-300, Hitachi, Japan). The samples were prepared on a carbon-coated copper TEM grid. Further, the surface characteristics were analysed using atomic force microscopy in contact mode on a multimode scanning probe microscope equipped with a Nanoscope IV controller at a scan rate of 5.086 Hz (Veeco Instruments, New York, NY, USA).

### 2.5. In Vitro Release Studies

The in vitro release of Dox from Dox-γG-AuNPs was studied at two different pH values, i.e., pH 5.3 (sodium acetate buffer saline) and pH 7.4 (phosphate buffer saline) as recipient dissolution media at 37 ± 0.5 °C. Dox-γG-AuNPs (10 mg/mL) was initially filled in the sac of a dialysis membrane (MWCO 5e6 kDa, HiMedia, Thane, India) to prepare nanoconjugate-loaded bags (1 mL in each membrane bag). These bags were then placed immediately into different receptor media (100 mL) for this mass transfer experiment. Aliquots of 1 mL were withdrawn at different time intervals while maintaining the sink conditions. Dox concentration was determined using a UV–Vis spectrophotometer (Varian Cary 50 Bio spectrophotometer, Agilent, Santa Clara, CA, USA) [32].

### 2.6. Cellular Experiments

#### 2.6.1. Cell Lines and Cell Culture

Rat C6 glioma cells were procured from ATCC. These cells were cultured in DMEM medium (Gibco, Mumbai, India) supplemented with 10% heat-inactivated fetal bovine serum (FBS; Gibco) and a 100× antibiotic-antimycotic solution (Gibco). The cell cultures were maintained at 37 °C in an environment with 5% CO_2_. 

#### 2.6.2. Cell Cytotoxicity

The MTT (3-(4,5-Dimethylthiazol-2-yl)-2,5-Diphenyltetrazolium Bromide) cytotoxicity assay is based on measurements of the activity of enzymes present in live cells that reduces MTT to give a purple colour. This assay was performed to determine the cytotoxicity of plain drug synthesised nanobioconjugates and their intermediates in various cancer cell types. C6 (rat glioma cancer cells) cells were plated in a 96-well plate (1 × 10^4^ cells/well) in Dulbecco’s modified eagle medium (DMEM) supplemented with 10% fetal bovine serum, 1% L-glutamine, penicillin antibiotics (100 IU/mL), and streptomycin (100 μg/mL) overnight. Treatment was given with Dox, AuNPs, γG-AuNPs and Dox-γG-AuNPs at different concentrations in media. The cells were incubated at 37 °C under an atmosphere containing 5% CO_2_ for 24 h, 48 h, and 72 h and then cell cytotoxicity was analysed using the standard MTT assay procedure. The relative cell viability (%) was determined by comparing the dissolved formazan’s absorbance with control wells containing only cell culture media [33].

#### 2.6.3. Live/Dead Analysis

Live/dead staining was conducted using a combination of Fluorescein diacetate (FDA; Invitrogen, Waltham, MA, USA), and Propidium iodide (PI; Invitrogen) solution. After the desired treatments, cells were exposed to a freshly prepared solution FDA (10 µg/mL) for 30 min and PI (50 µg/mL) for 15 min in a dark environment. Subsequently, the FDA/PI solution was carefully replaced with PBS to prevent the loss of dead cells during the media change. The cells were then promptly imaged using a fluorescence microscope (Nikon ECLIPSE Ti2, Melville, NY, USA).

#### 2.6.4. Cell Uptake of Dox

Intracellular uptake of loaded Dox from the synthesised Dox-γG-AuNPs was estimated in comparison to free Dox in C6 cells (seeded at 1 × 10^4^ cell concentrations each and incubated for 1 and 4 h). Free Dox (at 2.5 µg/mL) and Dox-γG-AuNPs at equivalent Dox concentrations, were added into each well and incubated for 1 and 4 h. Finally, the medium was removed, and cells were washed with PBS. The cells were fixed using 4% paraformaldehyde (PFA) and then stained with DAPI. Intracellular Dox uptake was analysed using a fluorescence microscope (Nikon ECLIPSE Ti2, Melville, NY, USA) [32,34].

#### 2.6.5. Cellular Uptake of Gold

The intracellular uptake of gold nanoparticles was determined using ICP-MS analysis as reported earlier. Briefly, the cultured C6 cells post treatment with nanobioconjugates were trypsinised at different time points. The cells were further lysed using triton X. A total of 100 µL of samples was digested using 5% HNO_3_:HCl (3:1) solution at 70 °C. The ion content was estimated by ICP-MS (Agilent technologies 7700 series, Santa Clara, CA, USA) using a standard ion solution (Tracecert standards).

#### 2.6.6. Cell Morphology Using SEM

The impact of treatments on the morphology of Rat glioma C6 cells was assessed using a scanning electron microscope (FESEM, ZEISS Supra 40VP, Jena, Germany) operated at 20 kV voltage. Following the respective treatments, the cells were first fixed with a 2.5% glutaraldehyde solution and subsequently dehydrated using a series of graded ethanol solutions. Prior to SEM analysis, the samples were allowed to air dry completely and were sputtered with gold before imaging.

#### 2.6.7. Intracellular ROS Detection

To quantify the levels of ROS produced in C6 cells under experimental conditions, a DCFH-DA (Dichloro-dihydro-fluorescein diacetate; Invitrogen, Waltham, MA, USA) assay was employed. In a brief overview, after subjecting the cells to the relevant treatments for 4 h, they were rinsed with PBS and subsequently exposed to 25 μM DCFH-DA for 30 min at 37 °C. Subsequently, immediate imaging of the cells was conducted using a fluorescence microscope. (Nikon ECLIPSE Ti2, Melville, NY, USA).

#### 2.6.8. qPCR Analysis

Total RNA was extracted from C6 cells that had been pre-incubated with Dox and nanobioconjugate using an RNeasy mini kit (Qiagen, Hilden, Germany), following the manufacturer’s instructions. Subsequently, first-strand cDNA was synthesised employing the Omniscript cDNA synthesis kit (Qiagen, Hilden, Germany). SYBR Green real-time PCR was carried out using a Quant Studio 3 system (Applied Biosystems, Waltham, MA, USA) in a total volume of 10 μL, with the iTaq Universal SYBR Green supermix (BIO-RAD, Hercules, CA, USA). Each reaction was carried out in triplicate, following the thermocycler conditions: one cycle at 95 °C for 1 min, succeeded by 40 cycles at 95 °C for 30 s and 60 °C for 1 min. To determine mRNA levels, relative quantification was performed utilising the standard curve method. Caspase expression levels were normalised to GAPDH for each sample. The presence of nonspecific amplification was verified through melt curve analysis.

### 2.7. Statistical Analysis

Quantitative data are expressed as mean ± SD (*n* = 3) and were analysed by use of Student’s t test. *p* values < 0.05 were considered statistically significant. A probability of *p* ≤ 0.05 was considered significant and *p* ≤ 0.001 was considered as extremely significant.

## 3. Results and Discussion

### 3.1. Synthesis, Characterisation of γ-Globulin Stabilized Gold Nanoparticles “γG-AuNPs” and Estimation of Hard Protein Corona

Size-controlled synthesis of gold nanoparticles was carried out using the Turkevich method. The reduction of gold salts into metal ions was carried out using sodium citrate as the reducing agent. The nanoparticles were washed and centrifuged and re-suspended in water. Synthesised AuNPs were collected and incubated with γ-globulin for protein corona formation. These protein-coated AuNPs were further washed with PBS and centrifuged to remove any free/unbound protein, resulting in a synthesis yield of 90.8 ± 3.1%. AuNPs were successfully synthesised with a hydrodynamic size distribution of 16.4 ± 0.4 nm with a PDI of 0.218 ± 0.013. Incubation with γ-globulin leads to the surface coating of AuNPs resulting in an increased average size of 18.3 ± 0.9 nm with a PDI of 0.274 ± 0.021. Zeta potential variations were analysed at different pH values (pH 5.5, 7.5 and 9) to estimate the variations in surface charge of AuNPs and γG-AuNPs. Investigation revealed that with the increase in proton [H^+^] concentration, the surface potential of naked and protein-stabilised AuNPs shifted towards the more positive side. But a significant difference can be observed at pH 5.5 (i.e., pH < pI of γ-globulin), where the surface potential of γG-AuNPs is on a much more positive side (7.21 ± 0.19 mV) when compared to plain AuNPs (Figure S1). The altered value of surface potential provides a strong confirmation of the essential colloidal stability of γG-AuNPs at biological pH values.

**Figure 1:**
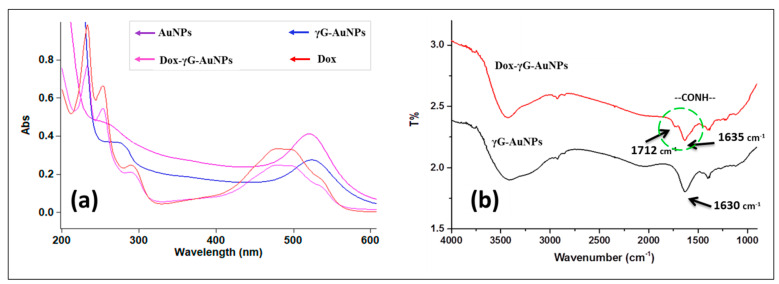
(**a**) UV–Vis spectroscopic data for Dox, AuNPs, γG-AuNPs and Dox-γG-AuNPs. (**b**) FT-IR spectra of γG-AuNPs and Dox-γG-AuNPs.

Figure 1a shows the UV–Vis absorption spectra of pure AuNPs and γG-AuNPs. The surface plasmon resonance (SPR) band at 524 nm corresponds to AuNPs. The bathochromic shift and broadened SPR band in the presence of γ-globulin suggests a change in the dielectric environment of AuNPs and hence conforms the strong conjugation of protein to AuNPs. 

Dox-γG-AuNPs nanobioconjugate was synthesised as per the above-mentioned procedure shown in Scheme 2. The conjugation of Dox to γ-globulin coating on AuNPs was obtained by carbodiimide coupling. The surface-exposed carboxylic group of Dox undergoes conjugation with EDC to form an unstable reactive ester and further addition of NHS results in the formation of a semi-stable amine-reactive NHS-ester. Afterwards, the addition of γG-AuNPs (that contains the free amino group of the exposed protein corona of AuNPs), to the activated Dox, leads to the formation of Dox-γG-AuNPs conjugate by a stable amide bond with a synthesis yield of 83.7 ± 1.5%. The UV–Vis spectra showed the nonappearance of a characteristic AuNP peak in Dox-loaded nanobioconjugate as a result of overlapping Dox spectra in the visible region in Figure 1a. A comparative FT-IR spectra of AuNPs and Dox-γG-AuNPs is shown in Figure 1b. The characteristic peaks of Dox, i.e., 3450 cm^−1^ (N-H), 3330 cm^−1^ (O-H), 2932 cm^−1^ (C-H), 1630 cm^−1^ (N-H), 1280 cm^−1^ (-C-O-C-) were observed in Dox-conjugated nanobioconjugate spectra. The observation of a peak shift from 1630 cm^−1^ to 1635 cm^−1^ and the presence of an additional peak at 1712 cm^−1^ (-C=O) after Dox conjugation was a result of amide (-C=O-N-H-) bond formation between the primary amine (-NH_2_) of Dox and the terminal carboxylic (-COOH) of surface-coated protein. Drug loading estimation (as per Equation (2)) revealed that 28.4 ± 1.32% of Dox loading was observed after EDC/NHS crosslinking. It was further calculated that 24.35 ± 0.64 µg of Dox was conjugated per milligram of Dox-γG-AuNPs nanobioconjugate (which is 2.43%). 

**Figure 2:**
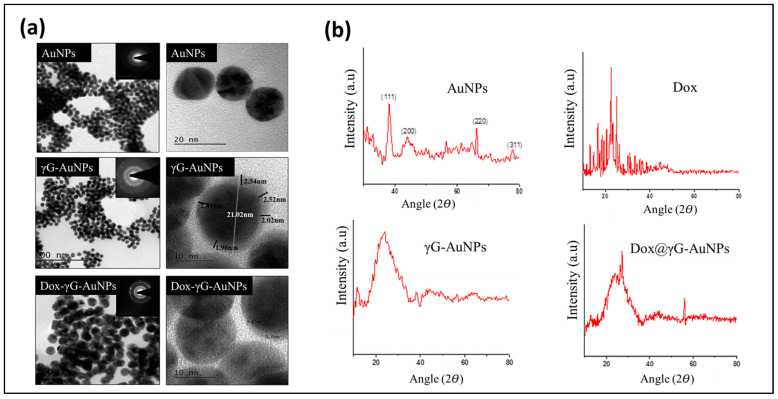
(**a**) TEM images of synthesised AuNPs, γG-AuNPs and Dox-γG-AuNPs (at different magnifications including SAED pattern in the inset). (**b**) XRD spectra for Dox, AuNPs, γG-AuNPs and Dox-γG-AuNPs.

TEM images show that AuNPs were synthesised in nanorange with an almost spherical shape and a narrow size distribution (Figure 2a). The average particle size has been estimated to be 17.5 ± 1.5 nm and the SAED pattern further confirmed the crystalline nature of synthesised AuNPs. TEM images confirm the presence of hard γ-globulin corona (average thickness of ~1.97 nm) on the nanogold surface. γ-globulin-stabilised AuNPs showed a predominately amorphous nature in the SAED pattern, with slight marks of a polycrystalline nature in the background. X-ray diffraction (XRD) patterns of AuNPs showed sharp and intense peaks (38.8° (111), 45.7° (200), 63.9° (220), and 76.4° (311) representing the crystallographic planes of face-centered cubic (fcc) AuNPs (Figure 2b). Surface-stabilised AuNPs showed a broad amorphous γG peak at a shorter 2θ range (18.23°–38.8°) referring to the diminished crystallinity of the nanostructures.

Interaction with the protein resulted in the development of two kinds of protein clouds around the nanometal surface viz. hard corona (essential to prevent colloid destabilization) and weak/soft/loose corona (hanging or accompanied protein molecules which form a secondary layer and are not necessary for the stabilisation process). As a result of electrostatic interactions between the positive surface of these AuNPs and the complementary charge on the amphoteric protein, a hard corona/shell was observed. Flocculation experiments confirmed no precipitation of γG-AuNPs in harsh conditions presented by saline phosphate buffer, presenting a straightforward protective effect of protein cloud on metal colloid stability. Protein estimation, i.e., BCA assay revealed the quantitative value of 0.137 µM of protein that stabilised the metal core (500 µg/mL) in the form of hard protein corona. 

Zeta size analysis reported a minimal increase in the hydrodynamic size (19.1 ± 0.7 nm) with a uniform distribution pattern, i.e., a PDI value of 0.218 ± 0.23, and a considerable shift in surface potential to −11.3 ± 0.6 mV was observed after the chemical conjugation of Dox using EDC/NHS chemistry. TEM images revealed that the surface of Dox-γG-AuNPs nanobioconjugate was still quite smooth with a slightly increased particulate diameter. SAED patterns further confirm the highly polycrystalline nature of Dox-γG-AuNPs nanobioconjugate because of the presence of crystalline Dox conjugated on the nanosurfaces. X-ray diffraction (XRD) patterns of synthesised conjugates suggest the presence of some Dox-specific crystalline peaks but with compromised intensity.

**Figure 3:**
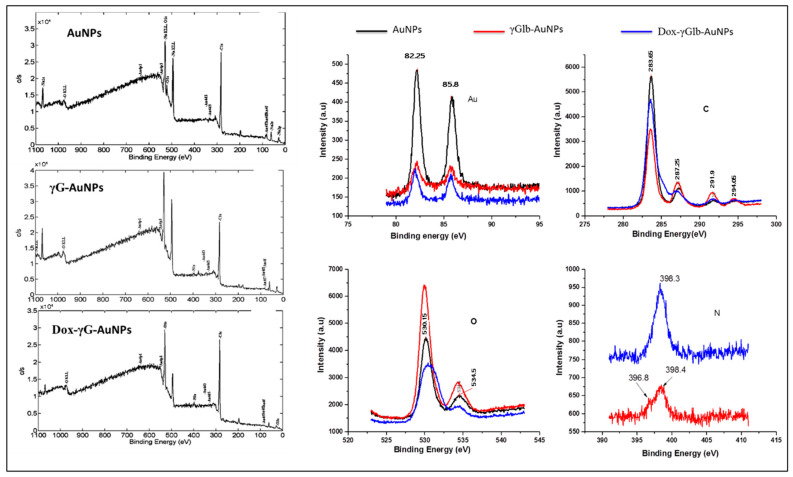
Complete X-ray photoelectron spectroscopy (XPS) spectra for AuNPs, γG-AuNPs, Dox-γG-AuNPs and high-resolution scans in the region of gold (Au), carbon (C), oxygen (O) and nitrogen (N).

The XPS scan of AuNPs, γG-AuNPs, Dox-γG-AuNPs and high-resolution scans in the regions of gold, carbon, oxygen and nitrogen are shown in Figure 3. High-resolution narrow scans from the gold “Au” region revealed the presence of doublet peaks at around 83.6 eV and 87.7 eV corresponding to the binding energies of Au, 4f_7/2_ and Au, 4f_5/2_, respectively. This explains the complete reduction of gold chloride into AuNPs with a zero valent state of gold. Dox conjugation results in the introduction of new peaks at around 285–287 eV “C” (after de-convolution of spectra, peaks represent C=O and C-N bond formation) because of a new amide linkage. Increased intensity of the C=O peak in the “O” spectra at around 530 eV was also observed after Dox conjugation. High-resolution narrow scans in the nitrogen region explain the presence of nitrogen as -NH_2_ and no other state was found.

### 3.2. In Vitro Release and Release Kinetics

**Figure 4:**
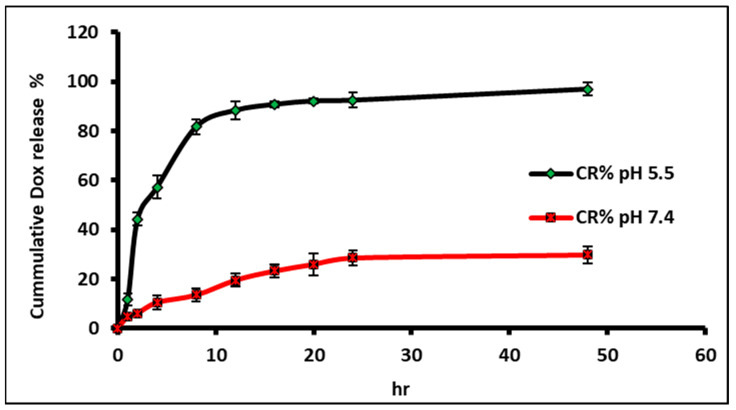
In vitro release of Dox at pH 5.5 and pH 7.4 from the synthesised Dox-γG-AuNPs.

In vitro release showed a pH-dependent controlled release profile for Dox at pH 5.5 and pH 7.4 from the synthesised Dox-γG-AuNPs nanobioconjugate. At pH 5.5, i.e., the tumour microenvironment, the Dox release followed a first-order pattern initially for 20 h (this is due to the drug being adsorbed or bound by ionic interactions) and afterwards, pseudo-zero order behaviour was observed. Different reports suggested that protein corona formation can reduce the burst effect of protein-conjugated nanoparticles or carriers with surface-loaded drugs [35,36,37]. This release rate can be modified and controlled by changing the corona composition, as seen in the case of Dox-conjugated γG-coated nanoparticles. After 48 h, the cumulative release observed for Dox at pH 5.5 was 96.9 ± 3.6% whereas at pH 7.4, the cumulative release observed was only 29.7 ± 4.6% (Figure 4). This may be attributed to the pH-responsive and acid-labile nature of the corona to which the Dox is conjugated. At pH 7.4, protein corona maintained its integrity due to a compromised solubility profile at the pH equivalent to its isoelectric point, and hence, very little of the drug is lost in systemic circulation (thus bypassing any toxic effects in the tissues/organs). But at a lower experimental pH, corona loses its inherent rigidity and provides more opportunity for Dox to be released from the surface. The release kinetics (Figure S2) were fitted in Pepas, Higuchi’s, zero- and first-order models. The results show that the drug release follows first-order kinetics at pH 5.5.

### 3.3. Cellular Experiments

#### 3.3.1. Cytotoxicity in Glioma Cells and Biocompatibility in Fibroblasts

**Figure 5:**
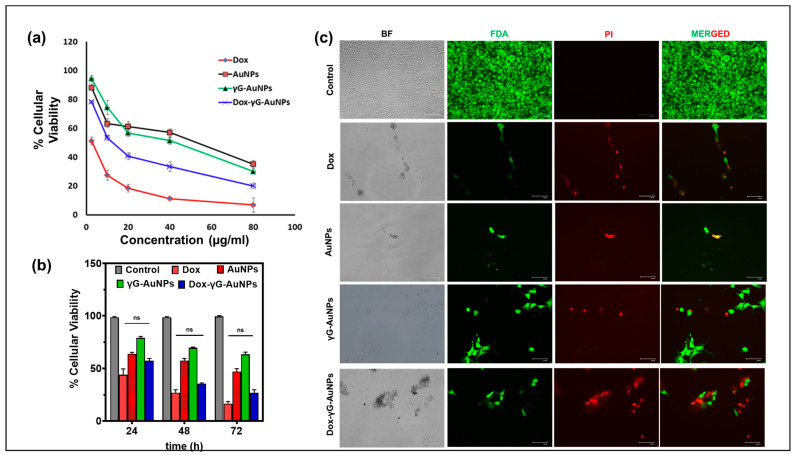
(**a**) Cell viability study of Dox, AuNPs, γG-AuNPs and Dox-γG-AuNPs in C6 cells, (**b**) Time-dependent cell viability of Dox, AuNPs, γG-AuNPs and Dox-γG-AuNPs at 10 µg/mL doses (ns: non-significant). (**c**) Live/dead analysis using FDA/PI post 24 h of treatment of Dox and Dox-γG-AuNPs. Scale bar represents 125 µm.

Cell cytotoxicity of all the samples was thoroughly assessed using a standard MTT assay on C6 cells (Figure 5a,b). The study showed a concentration-dependent cytotoxicity for all the tested samples, i.e., Dox, AuNPs, γG-AuNPs and Dox-γG-AuNPs with IC_50_ values of 2.67 ± 0.17 µg, 53.4 ± 2.61 µg, 42.12 ± 3.08 µg and 11.87 ± 2.44 µg, respectively. Toxicity shown by AuNPs and γG-AuNPs ensured that γ-globulin hard corona certainly helped the metal nanoparticles to cross the cell membrane without any prior entanglement with the media components, which eventually resulted in their increased cytotoxic potential [38]. Further, it was calculated that the concentration of Dox in Dox-γG-AuNPs is 2.43%, which comes around 0.27 µg. Henceforth, in terms of the equivalent IC_50_ Dox concentration, Dox-γG-AuNPs (0.27 µg) is 9.89 times (around 10-fold) more potent than plain Dox (2.67 ± 0.17 µg). In other words, treatment with Dox-loaded nanobioconjugate “Dox-γG-AuNPs” showed around a 10-fold increase in the cytotoxic potency in contrast to the equivalent concentration of plain Dox. This potentiation in anticancer activity can be understood as the dual attack of Dox along with protein-coated gold nanoparticles. Dox kills cancer cells using a very specific mechanism of DNA intercalation and inhibition of macromolecular biosynthesis whereas AuNPs provide a nonspecific attack of AuNPs (viz. DNA intercalation, free radical generation, promoting apoptosis and cell membrane damage etc.). Furthermore γ-globulin hard corona in Dox-γG-AuNPs ensures an uninterrupted and efficient uptake of nanobioconjugate inside cancer cells to display this combat attack. 

Biocompatibility was carried out in normal murine fibroblastic cells NIH3T3 that showed that the protein corona of gamma globulin over bare gold nanoparticles reduced their cytotoxic effect on normal cells. As seen in Figure S3, γG-AuNPs and Dox-γG-AuNPs showed better cellular viability in comparison to Dox and AuNPs. This shows that the pH-dependent release of a drug from the protein corona will spare the normal cells in comparison to the cancer cells. Gold nanoparticles have exhibited varying levels of activity across different cell lines, encompassing both cancerous and non-cancerous cell types [39]. These discrepancies in activity may be linked to the distinct characteristics of individual cell lines [40]. Nonetheless, the slight cytotoxicity observed in non-cancerous NIH3T3 cells when exposed to γG-AuNPs and Dox-γG-AuNPs raises concerns regarding the potential of these nanobioconjugates. Addressing this specific issue will necessitate the development of additional strategies to target the interaction of these nanobioconjugates, specifically with cancer cells.

#### 3.3.2. Live/Dead Analysis

As seen from the viability data, similar results were observed in the case of live/dead staining post 12 h of treatment. The treatments showed varying degrees of cellular damage to the cells when compared to the control cells that showed higher green fluorescence intensity. Bare gold nanoparticles and gamma globulin gold particles showed some damage to the cytoskeleton and the number of living cells was less than the control. Dox and Dox-γG-AuNPs showed extensive damage to the cellular matrix. Distinctive features of the apoptotic cell death pathway became apparent, including alterations in the cell membrane, disruption of the cytoskeletal framework, and nuclear damage. These changes lead to the complete breakdown of essential cellular structures, ultimately culminating in cell demise [41]. This was evident from the increased red fluorescence as seen in Figure 5c.

#### 3.3.3. Cellular Uptake of Dox

**Figure 6:**
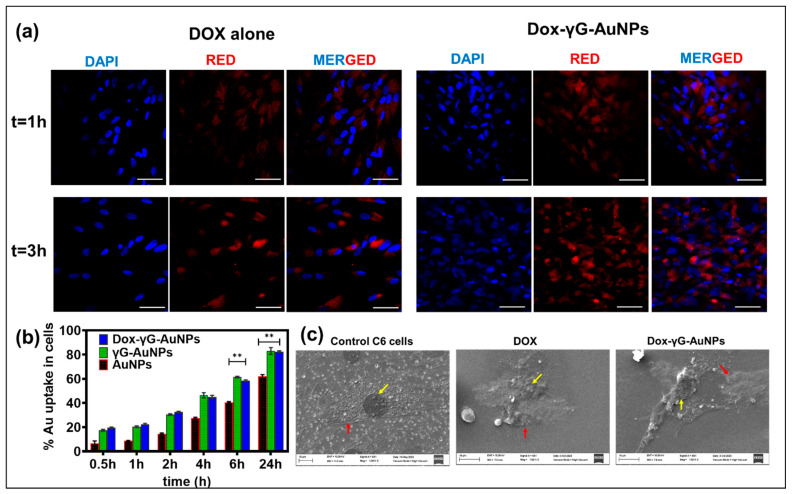
(**a**) Time-dependent cellular uptake of Dox from Dox-γG-AuNPs in C6 cells. DAPI was used to stain the nucleus. The scale bar refers to 40 µm. (**b**) Cellular gold concentrations of AuNPs, γG-AuNPs and Dox-γG-AuNPs in C6 cells. (**c**) Morphology of C6 cells post treatment using SEM analysis. The yellow arrows depict the nucleus, and the red arrows show the membrane (two-way ANOVA analysis using Tukey’s multiple comparison analysis, where *n* = 3, ** *p* ≤ 0.001).

Intracellular uptake of Dox from synthesised nanobioconjugate Dox-γG-AuNPs was studied using confocal imaging in comparison to the free Dox, as shown in Figure 6a. Confocal images after 4 h of treatment exposure showed a comparatively similar cellular uptake of Dox from Dox-γG-AuNPs in C6 cells. Results showed a strong correlation with the observed anticancer effects and also defined the role of γ-globulin surface corona. The intensity of Dox uptake from Dox-γG-AuNPs was only dependent on two factors: first is the uptake of preliminary released Dox in an acidic intercellular environment (i.e., exterior tumor microenvironment) and second is the direct uptake of 20 nm nanobioconjugate. Here, γ-globulin played a crucial role by providing a pH-responsive burst release in the very exterior of tumor cells which eventually gets absorbed into the cancer cells. Secondly, by virtue of the positive surface charge possessed by these nanobioconjugates (due to the γ-globulin behaviour in pH < pI), their cancer cell uptake becomes readily enhanced [42,43]. Hence, we can successfully state that the Dox-γG-AuNPs nanobioconjugate provides an increase in the cytotoxicity of cancer cells, where each component plays a defined role. 

#### 3.3.4. Cellular Uptake of Gold

The influence of the protein corona on the cellular uptake of nanobioconjugates was thoroughly examined through in-depth ICP-MS investigations. As illustrated in Figure 6b, the temporal patterns of uptake were scrutinised, revealing noteworthy distinctions. Pristine AuNPs exhibited a relatively slower cellular uptake, approximately 60 ± 1.8%, after a 24 h period. In stark contrast, γG-AuNPs exhibited a significantly higher uptake rate of 83 ± 1.5%, whereas Dox-γG-AuNPs showed a similarly enhanced uptake rate of 81 ± 2.3% during the same time frame. This pronounced enhancement in cellular uptake can be attributed to the presence of the γG coating on the bare AuNPs, which appears to facilitate more efficient endocytosis of these nanobioconjugates. Importantly, this heightened uptake was found to have a direct impact on cellular viability, as evidenced by comprehensive cytotoxicity assessments and live/dead analyses, indicating a potential avenue for therapeutic interventions.

#### 3.3.5. Cellular Morphology Using SEM

In this study, the impact of various treatment modalities on the cellular morphology of C6 glioma cells was investigated. Our approach involved a detailed analysis using scanning electron microscopy, shedding light on the structural changes induced by these treatments. As illustrated in Figure 6c, the control cells displayed an intact nucleus and a well-spread cytoskeleton, as indicated by the yellow and red arrows in the images. However, upon exposure to treatments involving doxorubicin (Dox) and Dox-γG-AuNPs, notable alterations in cellular structure became apparent. The cytoskeleton showed signs of damage, and the nucleus appeared shrunken and distorted. This observed morphological disruption aligns with our findings from cell viability and live/dead analyses. These complementary assays revealed extensive damage to the cellular membrane, ultimately leading to cell death within 24 h of treatment. These collective results provide valuable insights into the effects of the treatments on the structural integrity and viability of C6 glioma cells.

#### 3.3.6. Reactive Oxygen Species (ROS) Detection

**Figure 7:**
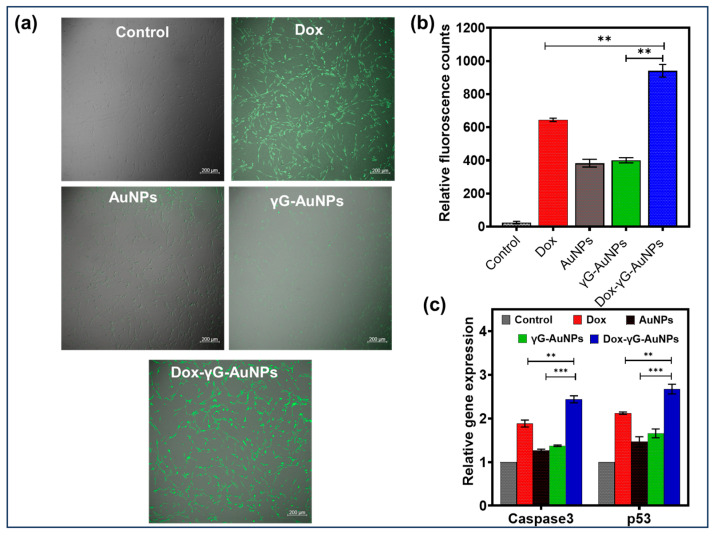
Intracellular ROS detection. (**a**) Fluorescence images. Scale bar is 200 µm. (**b**) Quantitative ROS analysis and Caspase upregulation; (**c**) qPCR analysis of Dox, AuNPs, γG-AuNPs and Dox-γG-AuNPs in C6 cells (two-way ANOVA analysis using Tukey’s multiple comparison analysis, where *n* = 3 ** *p* ≤ 0.01 and ***p ≤ 0.001).

Subsequently, we examined the intracellular generation of ROS in glioma cells using both fluorescence microscopy (Figure 7a) and quantitative analysis via the DCFDA method (Figure 7b) under identical conditions. The increase in ROS activity was seen in terms of green fluorescence of FDA in the cells. The fluorescence counts were higher for Dox and Dox-γG-AuNPs over bare AuNP and γG-AuNPs. Several studies have suggested a correlation between ROS generation and the disruption of the redox balance in cancer cells, leading to oxidative stress. Dox has been extensively utilised as a chemotherapeutic agent for various cancer treatments. Its mechanism of action is attributed to its capacity to bind to DNA-associated enzymes, intercalate within DNA base pairs, and target diverse molecular sites.

Recent research indicates that Dox can intercalate with both nuclear and mitochondrial DNA, resulting in a range of cytotoxic effects [44]. This results in the generation of highly reactive hydroxyl free radicals. Consequently, the concurrent application of doxorubicin and gold nanoparticles is anticipated to exhibit an increased ROS production, thereby promoting enhanced cancer cell apoptosis.

#### 3.3.7. qPCR

To gain deeper insights into the mechanisms underlying cellular death, we conducted a comprehensive analysis of the genes responsible for the p53 and caspase activation pathway. Caspases, key regulators of programmed cell death, function in a highly orchestrated sequence. The activation of Caspase 12 serves as the initial trigger, followed by the activation of Caspase 9, which, in turn, instigates the activation of the downstream “effector” Caspase 3 [45]. Our findings revealed significant changes in the expression levels of Caspase 3 and p53 in response to different treatments. Notably, we observed a robust upregulation of these caspases in cells treated with doxorubicin (Dox), with a twofold increase in Caspase 3 and a two-and-a-half-fold increase in Caspase 9 compared to control C6 cells (Figure 7c). In contrast, bare AuNPs and γG-AuNPs exhibited only marginal changes in Caspase 3 and p53 expressions, with approximately 0.5-fold and 0.6-fold increases, respectively, compared to control cells. These results emphasise the differential impact of various treatments on the activation of the caspase pathway. The findings from this study provide convincing evidence indicating that nanoparticles (NPs) exert a notable influence on p53-mediated cell death, and this effect appears to be closely linked to the modulation of endogenous ROS levels.

## 4. Conclusions

Our innovative approach to designing hybrid anticancer therapeutics culminated in the development of Dox-γG-AuNPs nanobioconjugates. These nanobioconjugates represent a bioinspired strategy that combines the strengths of gamma globulin protein corona pre-coating on the nanocore surface with efficient drug delivery mechanisms. This approach ensures unique surface stability for the nanocarriers, rendering them resilient to alterations induced by the composition of blood serum. From a drug delivery standpoint, we achieved efficient drug loading by chemically conjugating the model anticancer drug with protein-coated nanocarriers. Notably, our nanobioconjugates exhibited pH-sensitive drug release characteristics, promoting enhanced drug efficacy within the acidic microenvironment of cancer cells. In vitro anticancer studies demonstrated a remarkable increase in anticancer activity, attributed to the synergistic effects of doxorubicin (Dox) and gold nanoparticles (AuNPs). Dox exerts its specific mechanism of action through DNA intercalation and the inhibition of macromolecular biosynthesis, and AuNPs contribute to a non-specific attack, involving DNA intercalation, free radical generation, apoptosis induction, and cell membrane damage, among other mechanisms. Furthermore, our designed nanobioconjugate exhibited significantly improved in vitro cell uptake, cell toxicity and cellular damage via the caspase pathway in C6 cells compared to plain Dox. Moreover, our investigation delved into the intricate interplay between the p53-mediated ROS pathway and its pivotal role in the regulation of various cell fate outcomes, including apoptosis and necrosis. This pathway, which hinges on the delicate balance of ROS, emerges as a critical determinant in shaping cellular responses to external factors, thereby warranting further exploration to unravel its intricate mechanisms and implications and the underlying molecular mechanisms driving cellular responses to these treatments. In summary, the utilisation of the robust protein corona of γ-globulin on AuNPs proved highly advantageous for maintaining bio stability under challenging serum conditions and enhancing anticancer potential in pH-sensitive environments. This nanobioconjugate approach opens exciting prospects in the field of bio-inspired drug delivery. Concerns arise from the observed cytotoxicity in non-cancerous cells necessitating the development of targeted strategies for interaction with cancer cells. In essence, this approach holds promise for creating innovative anticancer hybrids by incorporating various drug molecules, and it presents prospects for precise modulation and targeting through specific ligand-based methodologies.

## Data Availability

Data are contained within the article and Supplementary Materials.

## Supplementary Materials

The following supporting information can be downloaded at: https://www.mdpi.com/article/10.3390/pharmaceutics16020208/s1, Figure S1: Zeta potential assessment of AuNPs, γG-AuNPs and Dox-γG-AuNPs at pH 5.5 7.4 and 9; Figure S2: Modelling the In-vitro release of Dox at pH 5.5 and in pH 7.4 from the synthesised Dox-γG-AuNPs; Figure S3: Biocompatibility of Dox, AuNPs, γG-AuNPs and Dox-γG-AuNPs in NIH3T3 cells post 24, 48 and 72 h (where *n* = 3).
