# Supplementary material for: Doxorubicin Conjugated γ-Globulin Functionalised Gold Nanoparticles: A pH-Responsive Bioinspired Nanoconjugate Approach for Advanced Chemotherapeutics
Source: Pharmaceutics. 2024 Jan 31;16(2):208. doi: 10.3390/pharmaceutics16020208 (PMC10893120; doi:10.3390/pharmaceutics16020208)
Supplement: Supplementary file 1 [file pharmaceutics-16-00208-s001.zip › pharmaceutics-2681651-supplementary.pdf]

Doxorubicin Conjugated γ-Globulin Functionalised Gold Nanoparticles: A pH-Responsive Bioinspired Nanoconjugate Approach for Advanced Chemotherapeutics

Gaurav Chauhan [1,*,†], Vianni Chopra [1,†], América García Alvarado [1], Jocelyn Alexandra Gómez Siono [1], Marc J. Madou [1,2], Sergio Omar Martinez-Chapa [1] and Manish M. Kulkarni [3,*]

1 School of Engineering and Sciences, Tecnologico de Monterrey, Av. Eugenio Garza Sada 2501 Sur,
Monterrey 64849, NL, Mexico; vchopra@tec.mx (V.C.); amealvarado-@hotmail.com (A.G.A.); mmadou@uci.edu (M.J.M.); smart@tec.mx (S.O.M.-C.)

2 Department of Mechanical and Aerospace Engineering, University of California Irvine, Irvine, CA 92697, USA

3 Centre for Nanoscience, Indian Institute of Technology Kanpur, Kanpur 208016, India

* Correspondence: gchauhan@tec.mx (G.C.); manishm@iitk.ac.in (M.M.K.)

† These authors contributed equally to this work.

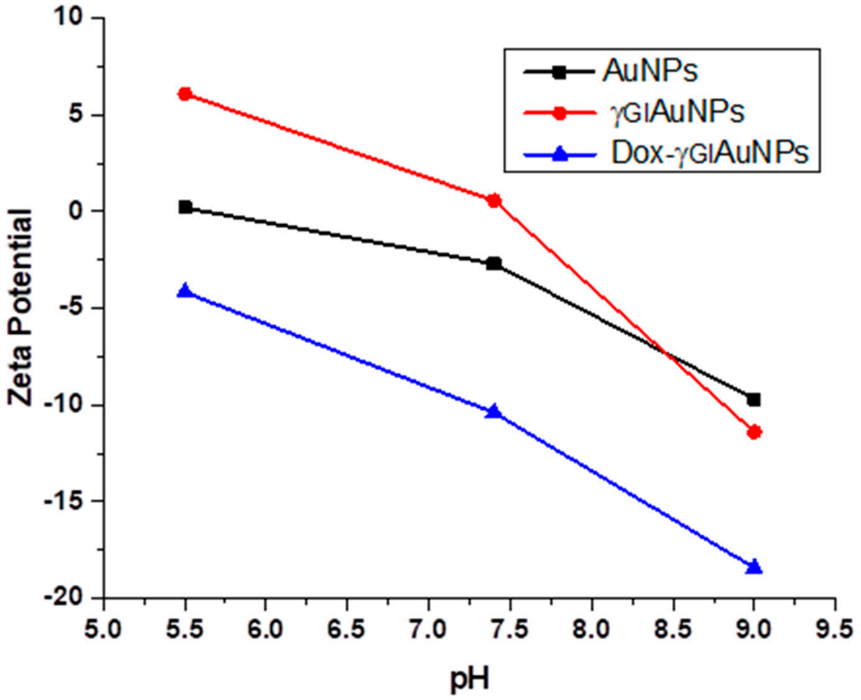

Figure S1. Zeta potential assessment of AuNPs, γG-AuNPs and Dox-γG-AuNPs at pH 5.5 7.4 and 9.

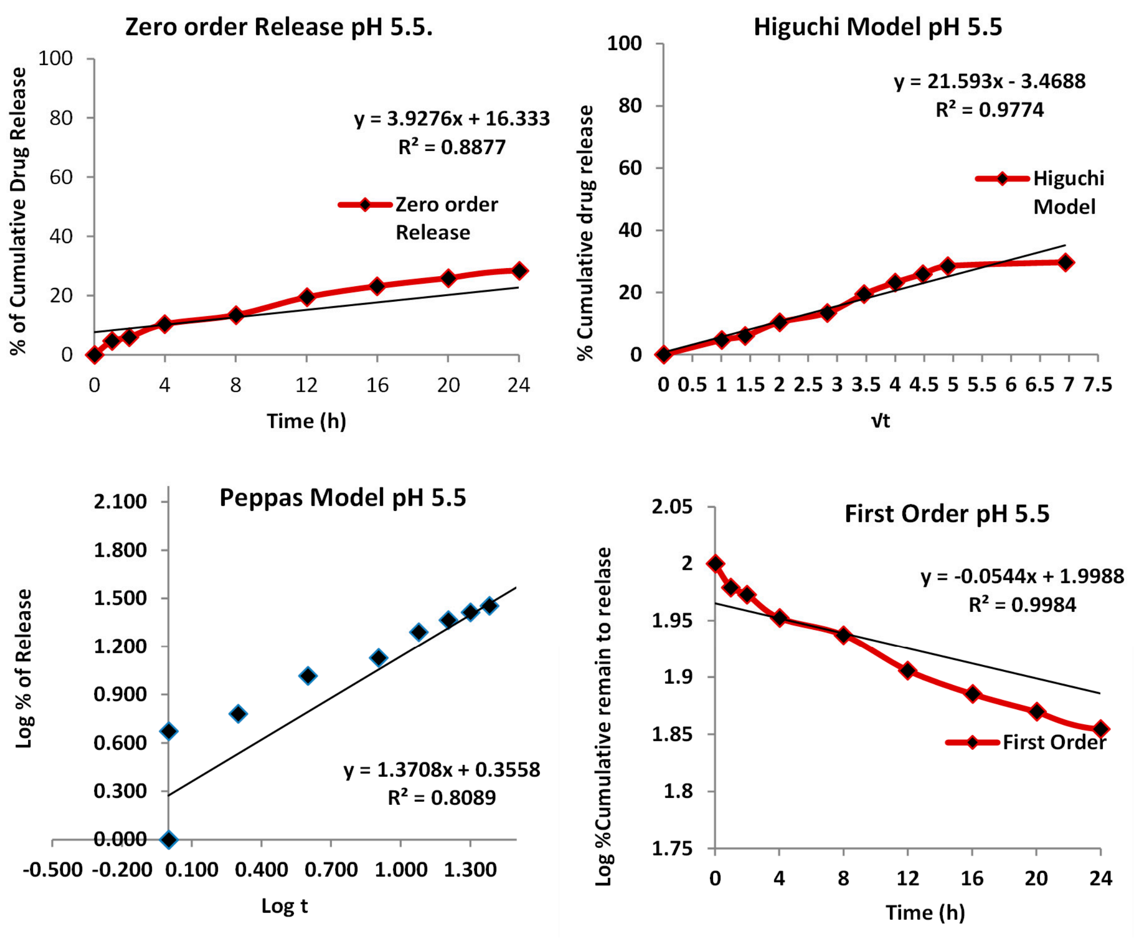

Figure S2. Modelling the In-vitro release of Dox at pH 5.5 and in pH 7.4 from the synthesised Dox-γG-AuNPs.

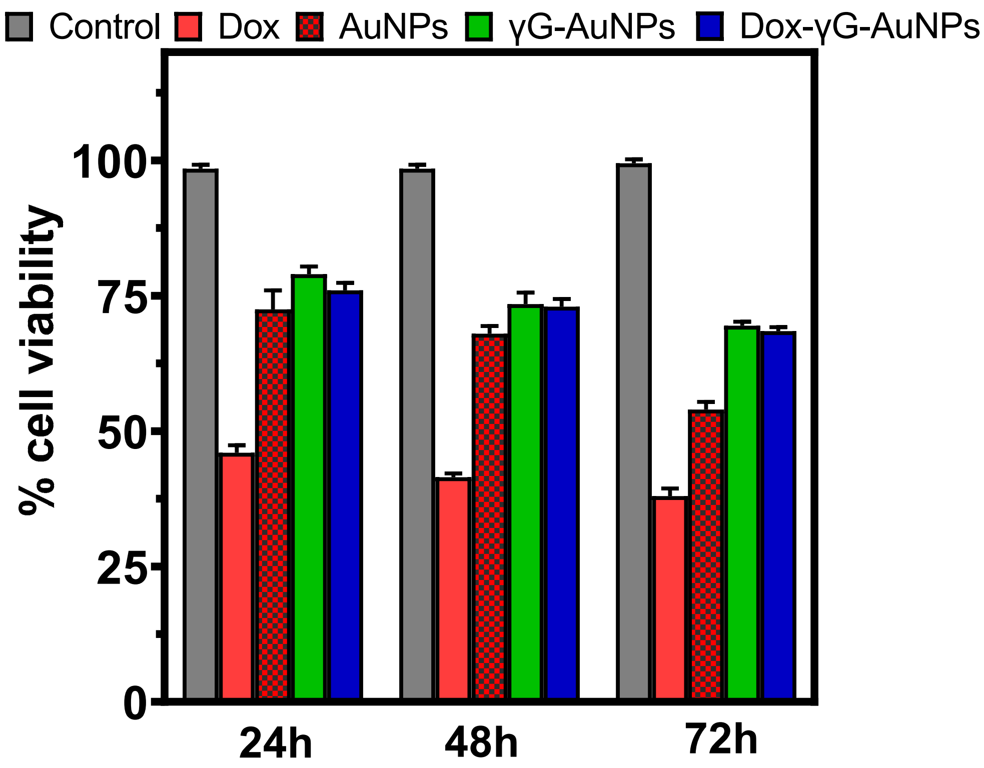

Figure S3. Biocompatibility of Dox, AuNPs, γG-AuNPs and Dox-γG-AuNPs in NIH3T3 cells post 24, 48 and 72 h. (where n = 3).
